# Gonococcal vaccines: Public health value and preferred product characteristics; report of a WHO global stakeholder consultation, January 2019

**DOI:** 10.1016/j.vaccine.2020.02.073

**Published:** 2020-06-09

**Authors:** Sami L. Gottlieb, Francis Ndowa, Edward W. Hook, Carolyn Deal, Laura Bachmann, Laith Abu-Raddad, Xiang-Sheng Chen, Ann Jerse, Nicola Low, Calman A. MacLennan, Helen Petousis-Harris, Kate L. Seib, Magnus Unemo, Leah Vincent, Birgitte K. Giersing, Gonococcal Vaccine PPC Expert Advisory Group

**Affiliations:** a World Health Organization, Geneva, Switzerland; b Skin and GU Medicine Clinic, Harare, Zimbabwe; c University of Alabama at Birmingham, Birmingham, AL, USA; d National Institute of Allergy and Infectious Diseases, Bethesda, MD, USA; e Centers for Disease Control and Prevention, Atlanta, GA, USA; f Weill Cornell Medicine-Qatar, Doha, Qatar; g Chinese Academy of Medical Sciences Institute of Dermatology, Nanjing, China; h Uniformed Services University of the Health Services, Bethesda, MD, USA; i University of Bern, Bern, Switzerland; j Bill and Melinda Gates Foundation, London, UK; k University of Auckland, Auckland, New Zealand; l Institute for Glycomics, Griffith University, Gold Coast, Australia; m Örebro University, Örebro, Sweden

**Keywords:** Gonorrhea, *Neisseria gonorrhoeae*, Vaccines, Gonococcal vaccines, Sexually transmitted infections

## Abstract

Renewed interest in developing vaccines against *Neisseria gonorrhoeae* has been sparked by the increasing threat of gonococcal antimicrobial resistance (AMR) and growing optimism that gonococcal vaccines are biologically feasible. Evidence suggests serogroup B *Neisseria meningitidis* vaccines might provide some cross-protection against *N. gonorrhoeae*, and new gonococcal vaccine candidates based on several approaches are currently in preclinical development. To further stimulate investment and accelerate development of gonococcal vaccines, greater understanding is needed regarding the overall value that gonococcal vaccines might have in addressing public health and societal goals in low-, middle-, and high-income country contexts and how future gonococcal vaccines might be accepted and used, if available. In January 2019, the World Health Organization (WHO) convened a multidisciplinary international group of experts to lay the groundwork for understanding the potential health, economic, and societal value of gonococcal vaccines and their likely acceptance and use, and for developing gonococcal vaccine preferred product characteristics (PPCs). WHO PPCs describe preferences for vaccine attributes that would help optimize vaccine value and use in meeting the global public health need. This paper describes the main discussion points and conclusions from the January 2019 meeting of experts. Participants emphasized the need for vaccines to control *N. gonorrhoeae* infections with the ultimate goals of preventing adverse sexual and reproductive health outcomes (e.g., infertility) and reducing the impact of gonococcal AMR. Meeting participants also discussed important PPC considerations (e.g., vaccine indications, target populations, and potential immunization strategies) and highlighted crucial research and data needs for guiding the value assessment and PPCs for gonococcal vaccines and advancing gonococcal vaccine development.

## 1 Introduction

Gonorrhoea, a sexually transmitted infection (STI) caused by the bacterium *Neisseria gonorrhoeae* (the gonococcus), has been a persistent public health problem for centuries. An estimated 87 million new cases occurred worldwide in 2016 [1]. Increased emergence of *N. gonorrhoeae* antimicrobial resistance (AMR) has heightened the possibility of future untreatable infections, and this threat to sexual and reproductive health (SRH) has increased global concern [2]. Untreated, or inadequately treated, gonorrhoea can lead to pelvic inflammatory disease (PID), infertility, adverse pregnancy outcomes, elevated risk for HIV acquisition and transmission, and ongoing transmission of *N. gonorrhoeae* to sexual partners and neonates. The World Health Organization (WHO) Global Health Sector Strategy on STIs has set targets for reducing gonorrhoea incidence by 90% by 2030 [3]. Recognizing that sustainable control of *N. gonorrhoeae* infections might not be achievable with current interventions, WHO’s strategy also highlights the crucial need for such new innovations for fighting this STI as effective vaccines. Interest in gonococcal vaccine development has been reinvigorated not only by an increasing global emphasis on vaccines in fighting AMR (see Box 1) [4], but also by observational studies indicating that vaccines developed for serogroup B *Neisseria meningitidis* might offer some protection against gonorrhoea, providing promise that gonococcal vaccines are biologically feasible [5].

Box 1 **Global public health strategies and initiatives relevant to gonococcal vaccines.**

**Decade of Vaccines and Global Vaccine Action Plan 2011–2020** [78], [79]
Global, collaborative initiative that includes a call for new research to expand the benefits of vaccines. A post-2020 plan “Immunization Agenda 2030” will be presented for endorsement by the World Health Assembly during 2020.
**Global Strategy for Women’s, Children’s and Adolescents’ Health (Every Woman, Every Child, 2015)** [80]
Prevention of sexually transmitted infections (STIs) (e.g., gonorrhoea) is part of global efforts for improving pregnancy outcomes, maternal and child health, adolescent health, and sexual and reproductive health.
**World Health Organization (WHO) Global Health Sector Strategy on STIs, 2016–2021** [3]
Gonorrhoea is 1 of 3 STIs prioritized for strategic global focus; 1 of 4 targets for 2030 is a 90% reduction in gonorrhoea incidence globally against a 2018 global baseline. In addition, STI vaccines are noted as key innovations needed for sustainable STI control.
**Global Roadmap for Advancing Development of Vaccines Against STIs** [6], [7], [77]
Collaborative global effort initiated by WHO and the National Institute of Allergy and Infectious Diseases (NIAID) that outlines crucial action steps for advancing vaccine development for STI vaccines, including gonococcal vaccines.
**WHO Global Action Plan on Antimicrobial Resistance (2015)** [81]
Development and use of new or improved vaccines that can prevent diseases that are becoming difficult to treat or are untreatable because of antimicrobial resistance (AMR) is a key area of focus.
**WHO Global Priority List of Antibiotic-Resistant Bacteria to Guide Research, Discovery, and Development of New Antibiotics (2017)** [37]
*Neisseria gonorrhoeae* is categorized as a high-priority pathogen for research and development efforts.
**WHO Global Action Plan to Control the Spread and Impact of Antimicrobial Resistance in *Neisseria gonorrhoeae* (2012)** [82]
Provides guidance regarding strategies for containing the spread of AMR in *N. gonorrhoeae* as part of broader national and international strategies for STI prevention and control.
**Vaccines to tackle drug resistant infections: An evaluation of R&D opportunities (Wellcome Trust and Boston Consulting Group, 2017)** [4]
Assesses the potential of vaccines to combat AMR for multiple pathogens; highlighted the strong case for advancing early research and development for *N. gonorrhoeae* vaccines.
**NIAID Workshop: Gonorrhoea** **Vaccines: the Way Forward (2015)** [83]
Workshop report outlines key preclinical and clinical development pathway considerations for gonococcal vaccines.

The Global STI Vaccine Roadmap outlines important action steps for advancing vaccine development for STIs, including gonorrhoea [6], [7]. WHO is coordinating key workstreams of the roadmap to evaluate the predicted global health, economic, and societal value of new STI vaccines and to identify vaccine attributes that can help optimize the value while vaccine candidates are still in early stages of development. To lay the groundwork for understanding the potential value of gonococcal vaccines and for developing gonococcal vaccine preferred product characteristics (PPCs), [8] WHO convened a global, multidisciplinary consultation in Geneva, Switzerland, in January 2019. The consultation included experts in gonorrhoea basic science, microbiology, epidemiology, clinical care, and public health control programmes, from low- and middle-income countries (LMICs) and high-income countries (HICs), along with experts in vaccine development and industry observers. The meeting was convened to discuss (1) the global public health need and goals for gonococcal vaccines and the value such vaccines might offer; (2) key considerations for gonococcal vaccine PPCs, in particular vaccine indications, target populations, and programmatic delivery approaches; and (3) vital research and data needs for building the value assessment and PPCs and advancing gonococcal vaccine development. This paper describes the main discussion points and conclusions from the meeting.

## 2 The need for gonococcal vaccines

### 2.1 *N. gonorrhoeae* infection and disease

*N. gonorrhoeae* typically infects urogenital, rectal, or oropharyngeal mucosae but can also cause conjunctival infection. Transmission of *N. gonorrhoeae* through penile–vaginal sex is efficient, with a substantial proportion of people becoming infected after a single exposure [9]. Among men, lower-genital tract infection typically manifests as urethritis, with purulent discharge or dysuria in the majority of cases. Among women, gonorrhoea primarily presents as asymptomatic or minimally symptomatic cervical infection that is often unrecognized or mistaken for other reproductive tract infections [10]. Coinfections, particularly with *Chlamydia trachomatis*, the causative agent of chlamydia, occur frequently [11]. *N. gonorrhoeae* can also be transmitted through oral–genital, genital–anal, and oral–anal contact [12]. Among both sexes, oropharyngeal and rectal infections are usually asymptomatic but can result in clinically apparent pharyngitis and proctitis, respectively.

The most common severe complication of gonorrhoea is upper-genital tract infection among women. Although data are limited regarding the proportion of *N. gonorrhoeae* cervical infections that ascend, 15% or more of untreated infections might ascend to cause PID, a common lower abdominal pain syndrome among young women [13]. Damage to the fallopian tubes from PID can lead to infertility, ectopic pregnancy, and chronic pelvic pain. Studies of women with documented PID suggest 15%–20% will have infertility later [14], [15], [16]. Gonorrhoea can also lead to infertility through subclinical or unrecognized PID [17]. Data regarding the consequences of PID have been collected primarily in HICs and have demonstrated that risk for sequelae is proportional to the delay in initiating treatment [18]. In areas with limited access to adequate health care for PID, a greater proportion of gonococcal infections might result in adverse outcomes. Ascending infection is now uncommon among men, primarily because infections are more likely to be symptomatic and promptly treated, but possible complications include epididymoorchitis and urethral stricture [10].

Gonorrhoea among pregnant women has been associated with risk for spontaneous abortion, intrauterine growth restriction, premature rupture of membranes, preterm birth, low infant birthweight, chorioamnionitis, and postpartum endometrial infection [19]. However, precise risks for each outcome have not been well-defined. Perinatal transmission of *N. gonorrhoeae* can result in neonatal conjunctivitis, a frequent cause of blindness before institution of topical ocular antibiotic prophylaxis for neonates globally [20], [21]. Additionally, epidemiologic and biologic data indicate a link between such inflammatory STIs as gonorrhoea with a 2- to 3-fold increased risk for HIV infection [22], [23], [24]. In the presence of *N. gonorrhoeae* infection, genital HIV RNA load increases, thus also increasing HIV transmissibility [25]. Rare complications of gonorrhoea include disseminated gonococcal infection, with bacteraemia and systemic involvement.

### 2.2 Epidemiology of *N. gonorrhoeae* infection and disease

WHO estimated the global prevalence of urogenital gonococcal infection to be 0.9% among women and 0.7% among men during 2016, with regional differences reflecting greater overall prevalence within LMICs [1]. Gonorrhoea prevalence ranged from 0.3% among both sexes in the WHO European Region to 1.9% among women and 1.6% among men in the African Region. However, regional estimates can mask wide variations by country. For example, general-population estimates of gonorrhoea prevalence have ranged from <0.1% during 2010–2012 in England [26], to 6.6% among women in South Africa [27], to >14% in antenatal clinics in Papua New Guinea [28]. The estimated global incidence rate, or new gonococcal infections, during 2016 was 20/1000 women and 26/1000 men, translating to 87 million (95% uncertainty interval: 59–123 million) new cases worldwide [1].

Prevalence and incidence of gonococcal infections vary both between and within countries and, in all settings, are likely to be higher among key populations, including men who have sex with men (MSM) and sex workers [9]. In a meta-analysis among studies of HIV preexposure prophylaxis (PrEP), gonorrhoea incidence among MSM was approximately 40/100 person-years [29]. Within HICs with low overall incidence, rates can be several-fold higher for racial, ethnic, or other marginalized minority populations with historical barriers to health care access [9], [30].

Additionally, recent surveillance data from several HICs reveal substantial and steady increases in gonorrhoea case reports where gonorrhoea prevalence and incidence had previously been low. For example, gonorrhoea case reports increased 83% in the United States during 2009–2018 [31] and 80% in Australia during 2013–2017 [30], and diagnoses increased 26% in just 1 year during 2017–2018 in England [32]. These increases have been greatest among adolescents and young adults and among MSM. Several health jurisdictions have observed an association between PrEP scale-up for HIV prevention and increases in gonorrhoea incidence [33]. Surveillance assessments within LMICs have been limited by less-developed infrastructure for testing and case reporting.

The global burden of gonococcal PID, infertility, ectopic pregnancy, and other adverse outcomes has not been quantified with precision but might be substantial. During the 1980s, a large study reported that approximately 85% of female infertility in sub-Saharan Africa was caused by tubal scarring from genital infection [34]. However, few studies have been conducted since then to quantify the burden of tubal factor infertility in different settings and the likelihood that gonorrhoea was a contributing factor. Many of the adverse outcomes of gonorrhoea can have multiple infectious causes, and lack of affordable diagnostic tests in LMICs limits routine etiologic assessment. Outcomes such as infertility and ectopic pregnancy might only be recognized years after infection.

### 2.3 AMR

Ever since antibiotics have been available for treating gonorrhoea, *N. gonorrhoeae* has demonstrated its ability to develop or acquire resistance rapidly to multiple antibiotics through different mechanisms [35]. *N. gonorrhoeae* is a WHO high-priority pathogen for research and development, because of increasing AMR to extended spectrum cephalosporins, the only remaining first-line monotherapy for gonorrhoea [2], [36], [37]. Monitoring for gonococcal AMR is performed at the global level through WHO’s Gonococcal Antimicrobial Surveillance Programme (GASP) laboratory network, which includes 67 participating countries [2]. During 2009–2014, 66% of participating countries reported isolates with decreased susceptibility to extended spectrum cephalosporins [36]. During 2015–2016, a total of 7 countries, primarily in WHO Western Pacific and Southeast Asian Regions, reported >5% of isolates with decreased susceptibility, or resistance, to ceftriaxone [2]. The majority of countries reporting to GASP are HICs, and data are lacking in many settings [2].

Verified clinical treatment failures with extended spectrum cephalosporins have occurred in several countries since the early 2000s [2], [38]. Additionally, one treatment failure with recommended ceftriaxone plus azithromycin dual therapy has now been confirmed [39]. Treating pharyngeal infections successfully with antibiotics is difficult, potentially because of reduced bioavailability in the oropharynx, resulting in more treatment failures relative to anogenital infections [2], [40].

### 2.4 Existing interventions for gonorrhoea management and control

Primary prevention for gonorrhoea consists of comprehensive sex education and condom promotion, which is essential but has had limited and unsustained success, particularly as condom use among MSM and other populations has decreased during the era of PrEP and other biomedical HIV prevention strategies [33]. WHO guidelines for gonorrhoea treatment recommend dual therapy with an extended spectrum cephalosporin plus azithromycin as a single dose [21]. Azithromycin also provides therapy for chlamydia, a frequent coinfection. In much of the world, gonorrhoea management involves a syndromic approach, using constellations of symptoms to guide STI treatment without diagnostic tests. This approach works relatively well for men, who are more likely to have symptoms indicative of gonococcal infection. In contrast, the vaginal discharge syndrome is poorly predictive of *N. gonorrhoeae* cervical infection [41], [42]. Accurate *N. gonorrhoeae* nucleic acid amplification tests (NAATs) exist but are expensive, can require laboratory infrastructure or equipment that is inaccessible in low-resource settings, and do not yield rapid results. Use of Gram stain microscopy can be a reliable, inexpensive diagnostic option for urethritis among men, but is not reliable among women or for extragenital sites.

Many gonococcal infections are asymptomatic or unrecognised but can still cause sequelae and be transmitted to sex partners and thus require screening for diagnosis and treatment. However, in most settings, even those with access to NAATs, screening is only recommended for groups at higher infection risk (e.g., MSM or sex workers), primarily because of cost and feasibility concerns. Several HICs with effective public health programmes that treated symptomatic infection, screened key populations, treated patients’ sex partners, and promoted condom use experienced substantial decreases in gonorrhoea incidence [43]. However, recent increases in gonorrhoea, even in settings with full access to available interventions, and the added threat of gonococcal AMR, highlight the fact that existing interventions are unlikely to be sufficient for controlling gonorrhoea. Thus, gonococcal vaccines might be the best hope for future sustainable control of *N. gonorrhoeae* infection.

## 3 Strategic public health goals for gonococcal vaccines

Meeting participants discussed overarching public health goals for gonococcal vaccines, considering the global epidemiologic context and existing interventions. They highlighted the need for gonococcal vaccines with the ultimate goals of (1) preventing adverse SRH outcomes and (2) reducing the impact of gonococcal AMR.

The experts noted the distinction between these goals and the more immediate objective of preventing *N. gonorrhoeae* infections. Prevention of infection is an outcome that can be easily measured in trials and facilitates achieving the ultimate goals. Further discussion will be needed for determining whether and how effects on SRH outcomes and AMR can and should be measured more directly in evaluating gonococcal vaccines, or whether measuring *N. gonorrhoeae* infection alone will be sufficient, along with bridging data to other outcomes.

The long-term goal of preventing SRH-related disease can be refined by specifying the highest priority *N. gonorrhoeae-*associated outcomes, considering such consequences as upper-genital tract morbidity among women (e.g., PID, infertility, ectopic pregnancy, or chronic pelvic pain), adverse pregnancy outcomes, increased HIV acquisition and transmission risk, ophthalmia neonatorum, and other effects on SRH. However, to prioritize specific outcomes, experts believe more data are needed regarding the precise global burden of the outcomes and the potential impact of AMR. For example, symptomatic urethritis among men might not be considered the most important health outcome from a relative morbidity and mortality standpoint. However, if gonorrhoea became untreatable because of AMR, gonococcal urethritis would become much more important.

Meeting participants emphasized the importance of including AMR in the strategic public health goals. AMR has fuelled the urgency for developing a vaccine and increased its value relative to other approaches. However, addressing AMR alone is insufficient as the only goal, because the value of reducing the impact of AMR stems from preventing the adverse SRH consequences of gonorrhoea. Meeting participants were unclear regarding how and when increasing AMR rates will translate into substantially reduced, or absent, clinical therapeutic options, but all agreed that gonococcal AMR represents a major global health threat. Given the unmet need for gonococcal vaccines in all countries, the aim is development of *N. gonorrhoeae* vaccines suitable for global use.

## 4 Feasibility of developing gonococcal vaccines

### 4.1 Gonococcal bacteriology and immune response

*N. gonorrhoeae* are Gram-negative diplococci and obligate human pathogens, which infect non-cornified epithelium in the cervix, urethra, rectum, oropharynx, and conjunctivae. Symptomatic infection is typically characterized by a neutrophil granulocyte-rich purulent discharge. Gonococci have evolved complex strategies for modulating and evading host innate and adaptive immune responses, including marked antigenic variability [44], [45]. Natural infection with *N. gonorrhoeae* does not appear to provide protection against future infection, even with the same strain, and repeated infections are common [45], [46]. Clinical trials with early gonococcal vaccines in the 1970s to early 1990s had disappointing results [45], and no surrogates or correlates of protection are known for human infection. Given these challenges, the feasibility of developing gonococcal vaccines had been in doubt for decades. Progress has since been fuelled by advances in whole-genome sequencing, proteomics, immunoproteomics, and molecular pathogenesis research, through which several new, stably expressed candidate antigens have been identified [44], [45]. New insights into the sophisticated mechanisms the gonococcus uses to evade the human immune system have also been revealed through studies with human immune cells and a mouse infection model [47]. Lack of protective immunity after infection might be related to induction of a Th17 response by *N. gonorrhoeae* that subverts development of a protective Th1 response [48]. Protection might also be impeded by antibodies against the surface protein Rmp that block the activity of antibodies against other surface molecules [49]. Gonococcal vaccines will need to induce a protective immune response that is superior to that generated during natural infection.

### 4.2 Serogroup B meningococcal vaccines and gonorrhoea

Optimism about the biologic feasibility of gonococcal vaccine development has been reenergized because of accumulating observational data related to vaccines developed for preventing disease from serogroup B *N. meningitidis*, a pathogen closely related to *N. gonorrhoeae*. After a mass vaccination campaign with a meningococcal group B outer membrane vesicle (OMV) vaccine in New Zealand (MeNZB), a large case-control study demonstrated that gonorrhoea case-patients were significantly less likely than control-patients to have been vaccinated [5]. The estimated vaccine effectiveness, after controlling for potential confounders, was 31% (95% confidence interval [CI]: 21%–39%). This effect appeared to be short-lived, and less reduction was observed for coinfections of *N. gonorrhoeae* and *C. trachomatis*. However, the findings were intriguing. A subsequent retrospective cohort study in New Zealand revealed that MeNZB vaccination was associated with a reduced rate of gonorrhoea-associated hospitalizations [50]. Although limited by small numbers, among those people vaccinated during adolescence, the hospitalization study estimated an adjusted vaccine effectiveness of 47% (95% CI: 18%–66%). Ecologic data from Cuba and a small observational study from Quebec also support apparent decreases in gonorrhoea incidence after use of OMV-based meningococcal vaccines [51], [52].

Several basic science studies have followed up on the observational data. OMVs are produced by *Neisseria* species during natural infection and *in vitro* culture and can be purified [53]. An 80%–90% nucleotide identity exists between *N. meningitidis* and *N. gonorrhoeae*, and several antigens present in meningococcal B OMVs are conserved in *N. gonorrhoeae* [54]. The New Zealand vaccine MeNZB is no longer available, but the MeNZB OMV antigen is a component of the licensed 4-component meningococcal serogroup B vaccine 4CMenB (Bexsero®, GSK). In one study, antibodies from people vaccinated with 4CMenB recognized gonococcal antigens, which might contribute to the predicted cross-protection of these vaccines [54]. In a recent study, 4CMenB accelerated clearance of *N. gonorrhoeae* in a mouse genital tract infection model [55]. With the observational data, these studies provide encouragement that gonococcal vaccines are biologically feasible.

### 4.3 Vaccine development efforts

Recent reviews have summarized the most promising antigenic targets, immunologic approaches, and current research and development efforts for vaccines against *N. gonorrhoeae* [7], [44], [45], [56]. A number of stably expressed conserved antigens might be promising gonococcal vaccine targets. The main vaccine approaches include meningococcal OMV vaccines, gonococcal OMV vaccines, a lipooligosaccharide epitope vaccine, and purified protein subunit vaccines [7]. Additional strategies involve formalin-inactivated whole-cell *N. gonorrhoeae*, virus-like particles, DNA or mRNAs [57]. Promising gonococcal vaccine candidates using these approaches are undergoing evaluation in animal models, with a variety of different antigen-delivery systems and adjuvants. Novel vaccine delivery systems include viral vectors, protein scaffolds, liposome preparations, nanoparticles or nanodiscs, and microarray patches [7], [45], [57].

Preclinical evaluation of gonococcal vaccine candidates is primarily conducted in mouse models with well-characterized features of female *N. gonorrhoeae* infection [44], [45]. Transgenic mice have been developed to alleviate some host restrictions, although mice cannot fully mimic human infection or disease [45]. A controlled human infection model with *N. gonorrhoeae* exists, but for safety reasons is limited to experimental urethral infection among male volunteers. The model is not widely available, and the window of study is 1–6 days before infection is treated [58]. Nonetheless, it can be used to measure antibodies, cytokines, and cell subsets upon infection and can provide a relatively rapid and less expensive way to conduct preliminary evaluations of vaccine candidates among humans. As of November 2019, no new gonococcal vaccine candidates were being evaluated in human clinical trials; however, clinical trials of licensed meningococcal group B vaccines for preventing gonococcal infections are planned for 2020.

## 5 Understanding the potential value of gonococcal vaccines

WHO assessments of the global value of vaccines aim to identify and articulate the potential value of a vaccine from the perspective of stakeholders in both LMICs and HICs, and for individual- and population-based health, economic, and societal benefits. Vaccines often take 12–15 years to develop at costs of ≥US$1 billion, and vaccine development programmes often fail [59]. To attract sufficient investment in gonococcal vaccine development, investors and vaccine developers will need to be convinced that gonococcal vaccines are technically feasible and commercially viable. However, beyond the economic investment case, WHO vaccine value assessments convey the range of effects a vaccine can have in meeting global public health and societal needs, which can guide policy and programme decision-makers in addition to vaccine developers and funders. Along with traditional economic assessments (e.g., cost-effectiveness analyses), such assessments consider overall reductions in disease incidence, impact on programmatic or health systems, and other social and economic benefits. Vaccine efficacy and disease burden need to be considered in the context of vaccine acceptability, affordability, and programmatic feasibility, and this can vary by setting. Thus, public health value of vaccines assessments also include country-level input and perspectives.

After reviewing the need and strategic public health goals of gonococcal vaccines and their technical feasibility, meeting participants reviewed models of potential vaccine impact, perspectives on gonococcal vaccines from different countries and regions, and other considerations for developing a global public health value assessment.

### 5.1 Modelling of predicted gonococcal vaccine impact

Mathematical modelling studies can contribute to multiple aspects of the value assessment and PPC process [60]. Modelling can be used to predict current and future trends in *N. gonorrhoeae* infection, disease, and AMR, and the potential impact of gonococcal vaccines [61], [62], [63]. The basic reproduction number (R_0_) for gonorrhoea, or maximum potential for a single infection to spread within a population, is believed to be low [64], indicating that a vaccine might not require high efficacy to have substantive population effects [65]. Two modelling studies predicted that, even with gonococcal vaccine efficacy of only 20%, lower than that observed in the New Zealand case-control study of serogroup B meningococcal vaccine [5], vaccination could result in a substantial decrease in *N. gonorrhoeae* infections [61], [63]. One model also predicted that vaccination can reduce the spread of gonococcal AMR [62]. Assuming all adolescents aged 13 years in a population received a nonwaning gonococcal vaccine with 50% efficacy, or a 100% efficacious vaccine waning after 7.5 years, modellers predicted that gonococcal infection prevalence might be reduced by 90% after 20 years [61]. Similar results were generated assuming only key population members were vaccinated, with 75% coverage [61].

Modelling has also been used to estimate gonorrhoea-associated costs. Direct medical costs of gonorrhoea cases in the United States were estimated at $162 million during 2008 [66]. Another model estimated that emerging ceftriaxone resistance might lead to 1.2 million more gonococcal infections over 10 years, costing $378.2 million [67]. Cost models from LMICs are limited, although estimates of implementation costs for the WHO Global Health Sector Strategy on STIs [3] includes costs of syndromic management of gonorrhoea for general populations and screening and treatment for key populations [68]. Intervention cost-effectiveness modelling is limited for all settings, particularly taking into account increasing AMR [63]. For the best predictive value, models require high-quality data inputs (see Section 7 for data needs).

### 5.2 Country and regional perspectives

Two panels of representatives from countries and regions provided perspectives about the need and potential public health value of gonorrhoea vaccines in the context of country- and regional-level epidemiology, AMR, and health care delivery, as well as local awareness, policy, and likely vaccine acceptability. The countries represented included Brazil, China, Kenya, South Africa, Thailand, the United States, and Zimbabwe. WHO Regional Advisors from the Americas and the Western Pacific Region and the European Centre for Disease Prevention and Control also provided input. Participants highlighted the widely varying prevalence of gonorrhoea between and within countries, which might affect the perceived value of vaccines in different settings. In the majority of countries, gonorrhoea prevalence is lower than that of the other common bacterial STI — chlamydia — but prevalence of both STIs can be high among certain populations (e.g., young women at high risk for HIV acquisition in sub-Saharan Africa) [11]. The increasing threat from gonococcal AMR is a concern across countries, given global transmission of AMR *N. gonorrhoeae* strains. Certain country representatives (e.g., Thailand’s) mentioned special considerations, including sex tourism, which can foster the global transmission of gonococcal AMR.

Lack of valid in-country prevalence and incidence data regarding gonococcal infection and AMR has hampered awareness and policy attention in many settings. STI care in the private sector and use of syndromic management can make data collection more difficult. Additionally, in some countries, a focus on HIV has distracted policymakers from investment in other STIs. However, growing awareness of AMR generally, recent increases in gonorrhoea and syphilis in HICs, and reports of treatment failures caused by gonococcal AMR have provided new opportunities for increased policy interest in gonococcal vaccines. In the United States, where infection and AMR surveillance data have been collected for years, drug-resistant *N. gonorrhoeae* is 1 of 3 pathogens considered to be a top-level urgent threat to be addressed by the government [69]. Some meeting participants questioned whether ministries of health might need to see clear trends of increasing morbidity from gonococcal AMR before allocating resources for gonococcal vaccines. Because gonorrhoea causes multiple types of morbidity but is typically not life-threatening, making the case for gonococcal vaccines to national policymakers can be more challenging. Cost-effectiveness data and a clear understanding of the adverse outcomes caused by gonorrhoea in local settings will be crucial for policy decision-making.

Although STI experts recognize the importance of controlling gonorrhoea given increasing AMR globally, awareness and knowledge of gonorrhoea and its potential disease outcomes among the public is relatively low in many countries [59]. For example, fertility is typically of substantial concern; however, the link to gonorrhoea and other STIs is not always recognized, and infertility prevention is not always prioritized by those allocating resources. Moreover, stigma regarding STIs, including gonorrhoea, is high and might be compounded in settings where a large burden of infection is among marginalized populations. That stigma can be a factor in acceptability of gonococcal vaccines. Human papillomavirus (HPV) vaccines are often promoted as anticancer vaccines rather than STI vaccines because of concerns regarding lower acceptability. Although populations are becoming aware of AMR, they might know less about gonococcal AMR specifically or believe they are not at risk. Some countries are focusing on youth health, which participants posited as an opportunity for positioning gonococcal vaccines because adolescents and young adults are particularly vulnerable to STIs.

### 5.3 Gonococcal AMR and other considerations regarding public health value

Meeting participants agreed that gonococcal vaccines are needed and would have value, but believed that available information is insufficient for fully quantifying the value and determining whether vaccine introduction is justified in all settings. A recent global report on AMR and vaccine development highlighted the strong case for advancing research and development for gonococcal vaccines because of high *N. gonorrhoeae* incidence, high morbidity, and circulation of AMR strains [4]. In addition to needing better, updated information about global gonococcal-related disease burden, a key consideration is how much gonococcal AMR is likely to increase, over what period, and how that might translate into treatment failures, lengthier and more costly treatment regimens, and increases in infection and disease outcomes. Another uncertainty is how best to measure and ascribe a value to the AMR threat and the potential role of vaccines in addressing it. Gonococcal AMR is only one part of a global problem. Antibiotic use or misuse for one infection can select for AMR in other pathogens, and reducing antibiotic use through a vaccine could reduce AMR for multiple pathogens [70]. WHO and other partners are developing a value attribution framework for vaccines against antimicrobial resistance for modelling these complex interactions [71].

The value of gonococcal vaccines will also need to be considered in the context of other available interventions, which might vary according to the status of AMR. At least 2 new antibiotics for gonorrhoea are in clinical trials, but this therapeutic area lacks strong commercial interest, and when a candidate will become available for clinical use remains unclear [72]. Additionally, *N. gonorrhoeae* has developed or acquired resistance to multiple classes of antibiotics over time. Additional interventions are also being studied (e.g., microbicides, oropharyngeal mouthwashes [73], and different packages of care) as are a range of novel AMR strategies, including antibodies, immune stimulants, and bacteriophages, which could all be reviewed within a value assessment [4].

## 6 Key considerations for gonococcal vaccine PPCs

Meeting participants discussed key considerations for gonococcal vaccine PPCs as a starting point for developing a formal WHO PPC document through a multistep consultative process [8]. The discussions focused on preferences for LMICs in addition to HICs to encourage development of gonococcal vaccines for global use.

### 6.1 Vaccine indications

Vaccine indications reflect the main prevention outcomes to be evaluated in vaccine clinical trials and provide the basis for licensure application. The choice of indication has implications for the feasibility of measuring outcomes and demonstrating efficacy and for eventual vaccine licensure and marketing. Prevention of *N. gonorrhoeae* infection is the vaccine indication that most easily addresses both overarching public health goals expressed by meeting participants: preventing *N. gonorrhoeae*-associated SRH morbidity and combating gonococcal AMR. Testing for infection can be performed easily and accurately in clinical trials, and reductions should translate into reductions in broader disease outcomes. However, demonstrating efficacy with a disease indication might be easier, because a vaccine might prevent disease to a greater extent than infection (e.g., by preventing *N. gonorrhoeae* ascension to the upper-genital tract rather than completely preventing infection). Conversely, a vaccine might disproportionately prevent infections that are less likely to cause sequelae. Disease endpoints and indications are often preferred by regulators (although not exclusively so) and might be easier to market. However, the choice of disease-related primary indications for gonococcal vaccines can be challenging. Among women, both symptomatic cervical infections and upper-genital tract infections (e.g., PID) are difficult to measure and confidently ascribe an aetiology. Further, even with prevention of disease, remaining asymptomatic infections could still propagate AMR. Although acquisition of AMR *N. gonorrhoeae* strains by individuals could be measured in vaccine trials, the likely benefit of vaccines in reducing the impact of AMR would be at a population level, because of reduced infections more broadly. Further review and discussion is needed for gaining final consensus regarding gonococcal vaccine indications, and new data (e.g., about correlates of protection or biomarkers of disease) might change the discourse.

### 6.2 Target populations

Wide variability in gonorrhoea epidemiology between and within countries guided discussion of preferred target populations for gonococcal vaccines. Meeting participants agreed that, in countries with relatively high prevalence of gonorrhoea among young sexually active populations, broad-based vaccination during early adolescence, aiming for presexual debut (e.g., the primary target age for HPV vaccination, ages 9–14 years) would be preferred [74]. More information might be needed, particularly cost-effectiveness modelling, to determine for how many countries or settings this target population for vaccination would be most appropriate, and according to which levels of prevalence, vaccine efficacy, and duration of protection. In areas where gonorrhoea prevalence among the general population is low but can be high among specific population groups, an alternative strategy would be targeting key populations. Certain groups (e.g., adolescent girls and young women in southern Africa and MSM) not only have higher gonococcal infection rates but are also at higher risk for HIV infection, which can be increased further during gonococcal infection. The global market for such population groups as MSM is likely to be relatively small, which will be a consideration for vaccine developers.

A focus on the period just before sexual debut is based on the need for peak protection during the time of highest incidence, which among general populations is typically during late adolescence and young adulthood. However, unlike HPV, whether people with prior exposure to *N. gonorrhoeae* might be more difficult to immunize than someone who is naïve to infection is unclear. Although induction of blocking antibodies to Rmp during natural infection might be a concern for OMV vaccines that contain this protein [49], this would not be a consideration for protein subunit vaccines or OMV vaccines in which the *rmp* gene is deleted. It is also possible that prior gonococcal infection might prime the immune system, resulting in a booster effect with vaccination. Duration of vaccine protection is a key consideration for determining the appropriate age at vaccination. Evidence regarding meningococcal serogroup B vaccines suggests possible cross-protection might be short-lived [5].

Meeting participants discussed whether the preferred target population should include both females and males. Because infection among both sexes can contribute to and be affected by AMR, both have disease consequences, and for general equity reasons, meeting participants favoured vaccination of both sexes. The most direct serious disease outcomes of gonococcal infection (e.g., infertility or adverse pregnancy outcomes) are among women, and single-sex vaccination can provide benefits for both sexes through herd immunity. WHO recommends HPV vaccination of adolescent girls only, on the basis of direct benefits for cervical cancer prevention and cost-effectiveness evaluations indicating limited incremental benefit of vaccinating adolescent boys among general populations if high coverage (>80%) is reached among girls [74]. Nonetheless, a number of countries (e.g., Australia, Brazil, the United States) have implemented HPV vaccination for boys as well. Further, MSM are often key populations for maintaining gonorrhoea transmission within HICs. Modelling and cost analyses considering differing scenarios for target populations and further information about the attributes of candidate gonococcal vaccines can help refine the discussion.

### 6.3 Programmatic delivery considerations

Programmatic delivery considerations are inextricably linked to intended vaccine target populations. Expanding global experience with HPV vaccination programmes makes reaching adolescent populations increasingly feasible, and new vaccines can be added to this platform, with shared delivery costs. Countries delivering HPV vaccine only to adolescent girls might need to adjust their programmes if gonococcal vaccines are recommended for both sexes. Vaccination programmes targeted to key populations have been difficult to implement in the past [75]. However, expansion of PrEP and other HIV prevention programmes to key populations might provide more efficient platforms for targeted vaccination. Further, if gonococcal vaccines have adequate efficacy among those with prior infection, or even a booster effect, administering the vaccine to people with diagnosed gonorrhoea, or their partners, would be a tailored way of finding those at highest risk.

Vaccine acceptability and coverage rates vary by country and vaccine. Many countries achieve excellent coverage rates for most vaccines and vaccines are highly acceptable. Other countries are, however, increasingly affected by vaccine hesitancy. Demonstration of safety and efficacy is always essential, but other factors can contribute to vaccine hesitancy, particularly for STI vaccines. Hepatitis B virus and HPV are STIs, but these vaccines are not promoted as STI vaccines but rather as hepatitis/hepatic cancer– and cervical cancer–prevention vaccines, respectively. Promoting gonococcal vaccines as anything other than STI vaccines might be difficult. Although a vaccine that helped preserve fertility or healthy pregnancies might be acceptable, these gonococcal disease outcomes have many possible causes, and the contribution of *N. gonorrhoeae* will be harder to evaluate in trials and thus to promote directly.

Combination vaccines are another consideration. Many practitioners believe that to target PID and other infectious reproductive-tract conditions, a dual gonococcal-chlamydial vaccine is desirable [59]. Both infections are STIs with similar sites of infection and disease outcomes, although their immunobiology and epidemiology have key differences. Development pathways are typically more straightforward and less resource-intensive for stand-alone vaccines than for combination vaccines. These products are often developed and evaluated separately, then combined. However, licensed serogroup B meningococcal vaccines might demonstrate some cross-protection against gonorrhoea, which could provide a short-cut to a combined meningococcal-gonococcal vaccine. Incidence of the different serogroups of meningococcal infection, and thus recommendations for serogroup-specific meningococcal vaccines, vary widely across countries and populations [76]. Meningococcal serogroup B vaccines are recommended in a relatively limited number of countries, primarily HICs. A meningococcal-gonococcal vaccine might be used more broadly. Trials directly evaluating the efficacy of licensed meningococcal serogroup B vaccines against gonorrhoea will provide information for determining the potential for combined versus stand-alone gonococcal vaccines.

## 7 Research and data needs

Meeting participants identified research and data needs that are crucial for assessing the public health value and for defining PPCs for gonococcal vaccines. Table 1 displays these research and data needs according to the key activities outlined in the Global STI Vaccine Roadmap, as follows: (1) obtaining better epidemiologic data regarding infection, disease, AMR, and natural history; (2) modelling predicted infection, disease, and AMR trends, economic burden, and theoretical vaccine impact and cost-effectiveness; (3) advancing basic science, translational, immunobiologic, and clinical research; and (4) encouraging investment and planning for policy and implementation decisions in advance [6], [7], [77].

**Table 1:** Gaps in knowledge and data and research needs for gonococcal vaccine development.

Area	Data gap or research need	Notes and additional considerations
*Obtaining better epidemiologic data regarding Neisseria gonorrhoeae (Ng) infection, disease, antimicrobial resistance (AMR), and natural history*		
Infection	• Improve global and regional estimates of Ng infection • Obtain prevalence and incidence data regarding Ng infection from more settings and populations • Evaluate overlap of Ng infection epidemiology with that of meningococcal serogroup B infection and introduction of meningococcal B vaccines across countries	• Development and validation of cheap, feasible Ng diagnostic tests is crucial for obtaining better data, especially for low- and middle-income countries (LMICs) [84] • Conduct strategically determined, additional prevalence studies in selected LMIC areas where most data are being imputed • The World Health Organization (WHO) has published a standard protocol for conducting gonorrhoea prevalence surveys in antenatal settings [85] • Use of existing Ng infection data from clinical trials (e.g., HIV prevention, human papillomavirus (HPV) vaccine, maternal studies) should be explored

Disease	• Improve global and regional estimates of Ng-associated clinical and disease outcomes • Obtain prevalence and incidence data regarding Ng-associated disease from more settings and populations, particularly in LMICs • Determine attributable fractions of such outcomes as PID and tubal factor infertility (TFI) caused by gonorrhoea	• Outcomes can include urethral discharge, pelvic inflammatory disease (PID), infertility, ectopic pregnancy, chronic pelvic pain, adverse birth outcomes, ophthalmia neonatorum and other eye disease, Ng-associated HIV infection, and other outcomes (e.g., epididymitis, proctitis, disseminated gonococcal infection, or male infertility) • Systematic reviews can be conducted first to summarize what is currently known about overall disease burden (e.g., all infertility or all TFI), about Ng-associated disease burden (e.g., Ng-associated PID and infertility), and about the methods used for attributing etiology to Ng (e.g., Ng serologic tests) • Updated etiologic studies of PID using cervical testing and of the proportion of infertility that is tubal factor [34], are needed in diverse settings • Improved methods for measuring upper-genital tract disease (e.g., biomarkers, radiology, or case definitions) and for measuring past Ng infection (e.g., improved serologic tests) would be valuable • Explore potential data sources regarding gonorrhoea-associated adverse pregnancy outcomes

AMR	• Obtain more globally representative assessments of Ng AMR, transmission, and clinical treatment failures • Determine trends in Ng AMR in more settings and among more populations	• Increase number of countries testing gonococcal isolates for AMR and reporting through WHO’s Gonococcal Antimicrobial Surveillance Programme (GASP) [2] • Improved diagnostic tests, including rapid tests for Ng AMR would aid evaluation • Systems are needed for identifying and reporting clinical treatment failures for gonorrhoea and other key AMR metrics (e.g., sentinel surveillance sites in LMIC settings)

Natural history and transmission	• Improve understanding of the proportion, predictors, and timing of Ng cervical infections ascending to the upper-genital tract, causing PID and resulting in long-term sequelae • Gain insight into the factors associated with acquisition, transmission, and duration of infection, including at multiple anatomical sites	• Innovative, ethical study designs are needed for assessing the natural history of Ng infection • Explore study designs used for understanding chlamydia natural history and their role in evaluating gonorrhoea (e.g., evaluating rates of PID in the interval between testing and treatment [86] or using serial specimens from existing prospective studies) • Couples studies might help researchers understand transmission from different anatomical sites and other factors like bacterial load

*Modelling gonococcal infection, disease, AMR, economic burden, and theoretical vaccine impact and cost-effectiveness*		
Overall	• Review and summarize the models that have been published, are ongoing, or are planned • Determine priority data needs for robust modelling • Prioritize and coordinate modelling efforts across groups, initiatives, and interventions	• Modelling efforts related to gonorrhoea across different initiatives (e.g., AMR) and interventions (e.g., new antibiotic development) will need to be coordinated to increase model efficiency and utility • Strengthened data on burden of infection, disease, AMR, transmission, and natural history is important for all models • Consensus regarding a plausible range of important model assumptions will be valuable • Model comparisons can strengthen robustness of conclusions from modelling studies

Models of Ng infection, disease, AMR, and economic burden	• Develop dynamic models of Ng infection, disease, and AMR in varied settings • Estimate global and regional economic burden of Ng infection and disease • Predict future trends in Ng infection, disease, AMR, and costs	• Improve data for modelling inputs and assumptions, including information about key populations and sexual networks • Modelling can be difficult for low prevalence infections with heterogenous distribution within the population • Predictions of the effect of increasing AMR on infection and disease incidence and on costs can help refine the value of a vaccine

Vaccine impact models	• Model the potential effectiveness of a future Ng vaccine in the context of the observed epidemiology and disease burden in different settings • Model potential vaccine impact against different assumptions and scenarios	• How to value AMR, a vaccine’s potential effect on AMR, and a vaccine’s impact in the context of AMR will be key to understanding overall vaccine impact and value • Understanding vaccine impact considering different target populations, immunization strategies, and efficacies can guide PPCs in addition to value propositions • Models should consider both high-income countries (HICs) and LMIC settings • Will be important to include alternative interventions (e.g., new antibiotics) in models

Cost-effectiveness models	• Model the potential cost-effectiveness of a future Ng vaccine given the observed and predicted health and economic burden in different settings • Model potential cost-effectiveness against different assumptions and scenarios	• Systematic reviews can assess what is known about gonorrhoea health care–seeking and –usage, and costs of care and treatment, for both infection and disease • Work will be needed to refine estimates of disability adjusted life-years and quality adjusted life-years considering all Ng outcomes • Cost-effectiveness analyses can guide preferred product characteristics (PPCs) in addition to value assessments

*Advancing basic science, translational, immunobiologic, and clinical research*		

Experimental systems	• Refine animal models and *in vitro* systems to expand the range of features of human infections that can be studied and to enable vaccine candidate assessment • Explore and optimize use of controlled human infection models (CHIMs) to study Ng immune responses and vaccine efficacy	• Continued understanding of complex Ng immunobiology can lead to new target antigens, novel adjuvant, or delivery systems • Improved preclinical systems for evaluating vaccine candidates can facilitate entry into clinical evaluation • Better data about pathogenesis and immunity in human infections can be compared with animal models to refine them • Current CHIMs exist only for male urethral infection, but could be developed for others (e.g., postmenopausal women)

Antigen discovery and vaccinology	• Continue to take advantage of new technologies to screen for new antigenic targets and define the most promising list of candidates [7], [44], [45], [56] • Further define mechanisms of immunity and immune evasion, which could help in developing adjuvants and delivery platforms for Ng vaccines • Evaluate vaccine candidates in preclinical models or CHIMs	• Evaluation of vaccine candidates is challenging, given that no established surrogate markers or correlates of protection against Ng exist • Given antigenic variability of Ng, a combination of antigens might be needed to provide broad protection against different Ng strains • Findings related to meningococcal outer membrane vesicle (OMV) vaccines and possible cross-protection for Ng can provide direction for vaccine development

Translational, immunobiologic, and clinical studies	• Conduct studies to obtain better data on human immune responses to Ng infection, including prospective evaluations of responses associated with reinfection • Evaluate the effect of licensed meningococcal serogroup B OMV vaccines on Ng acquisition • Evaluate the effect of coinfections, the microbiome, and hormonal status on Ng infection, disease, and immune response • Better understand the role of oropharyngeal and rectal infection in Ng transmission and promotion of AMR • Facilitate progression of promising preclinical candidates into clinical evaluation as soon as possible	• Clinical studies are needed to examine host factors and immune responses during Ng infection, and those predicting the likelihood of infection, reinfection, or ascension to the upper-genital tract • Innovative studies can be modelled on those conducted for chlamydia (e.g., studies reporting that clearance of infection between testing and treatment is linked with reduced risk for repeat infection) [87] • People with an increased risk for complicated Ng disease from complement disorders can provide clues to correlates of risk and protection [88] • Efficacy of meningococcal serogroup B vaccines ideally will be evaluated through clinical trials specifically designed for examining efficacy against Ng infection or disease acquisition; prospective observational studies as the vaccines are rolled out in new areas can also add insight • Couples studies might be useful for evaluating transmission and factors associated with transmission • Correlates of protection might be difficult to identify but would be highly useful; although not essential for vaccine development and licensing, they can make bridging studies easier

*Encouraging investment and planning for policy and implementation decisions in advance*		

Value assessment and PPCs	• Consolidate data on burden of disease, economic burden, and vaccine impact and cost-effectiveness • Understand drivers of gonococcal vaccine development and who the main stakeholders are • Obtain country-level input regarding the potential value of Ng vaccines and features of a vaccine that would be essential and those that would be desirable in different settings	• Improving the quantity and quality of underlying disease data is crucial for developing the vaccine value assessment and PPCs • Considering the interests and needs of different stakeholders is vital (e.g., WHO’s Strategic Advisory Group of Experts, Gavi, the Vaccine Alliance, funders, vaccine developers, national policymakers, health care providers, individuals at risk, parents, and civil society) • Value-of-vaccines assessments consider more than just health benefits and can also include broader societal and public value

Acceptability and implementation	• Evaluate the level of awareness and knowledge about gonorrhoea and Ng AMR and risk perception among young people and their parents, health care providers, and policymakers • Obtain country-level information regarding the likely acceptability of and demand for Ng vaccines from a broad range of countries • Evaluate the potential acceptability of Ng vaccines and potential barriers to acceptance or uptake by individuals and communities for different populations and settings • Assess the potential delivery systems for gonococcal vaccines for different target populations in varied health care systems	• Increasing awareness of Ng AMR and treatment failures can affect both awareness and demand for Ng vaccines • Understanding potential acceptability of Ng vaccines and how they would be used, including country-level input, early in vaccine development can help guide development of vaccines that are suited for global use and able to be implemented more quickly upon licensure and pre-qualification

## 8 Conclusion

Public health interest in developing vaccines for gonorrhoea has grown dramatically in recent years, given increasing gonococcal AMR and promising evidence that gonococcal vaccines might be biologically feasible [2], [5]. The 2019 WHO global stakeholder consultation on gonococcal vaccines focussed on the potential value of gonococcal vaccines, the characteristics they should have to optimize their global public health value, and how such vaccines might be used, if available. Meeting participants emphasized that, with respect to broad public health goals, gonococcal vaccines should address both the disease outcomes of gonorrhoea and the increasing threat of gonococcal AMR. To better understand the full potential benefit that gonococcal vaccines can have in reaching these goals, the experts stressed the need to fill several data gaps. These gaps include more precisely quantifying the magnitude of *N. gonorrhoeae*-associated disease burden for such outcomes as infertility and modelling the predicted role of gonococcal vaccines in controlling gonococcal infections in the context of AMR or in reducing the impact of gonococcal AMR more directly. These data will be crucial for assessing the global health, economic, and societal value of gonococcal vaccines, which can justify investment and aid decision-making about future vaccine policy and programmes. Meeting discussions concerning gonococcal vaccine indications, target populations, infection and disease endpoints, and relevant vaccination strategies provide the basis for comprehensive PPCs for gonococcal vaccines to encourage development of vaccines suitable for both LMIC and HIC contexts. These epidemiologic, programmatic, and policy considerations should be addressed in parallel with advancing preclinical and clinical research and development, including direct assessment of the ability of meningococcal serogroup B OMV vaccines to prevent gonorrhoea. These activities, together, can help catalyse development of viable gonococcal vaccines and realize a long-awaited innovation in the control of an important STI.

## Declaration of Competing Interest

Drs Gottlieb, Ndowa, Hook, Deal, Bachmann, Abu-Raddad, Chen, Low, MacLennan, Petousis-Harris, Unemo, Vincent, and Giersing report no conflicts of interest. Dr Jerse’s laboratory has conducted or is conducting research with the following companies under cooperative research and development agreements (CRADAs) or subcontracts to National Institutes of Health (NIH) grants: Chiron Corporation; Discuva, Ltd; GlaxoSmithKline; Microbiotx, Inc; The Population Council; Reoxcyn Discoveries Group; Tetraphase, Inc; Topcaid; VenatoRx; and Yaso Therapeutics. Dr Seib reports a brief consultancy with GlaxoSmithKline to provide expert advice on gonorrhoea in Australia, which occurred after the WHO Consultation in January 2019.

## References

[1] Rowley J., Vander Hoorn S., Korenromp E., Low N., Unemo M., Abu-Raddad L.J. (2019). Chlamydia, gonorrhoea, trichomoniasis and syphilis: global prevalence and incidence estimates, 2016. Bull World Health Organ.

[2] Unemo M., Lahra M.M., Cole M., Galarza P., Ndowa F., Martin I. (2019). World Health Organization Global Gonococcal Antimicrobial Surveillance Program (WHO GASP): review of new data and evidence to inform international collaborative actions and research efforts. Sex Health.

[3] World Health Organization. Global health sector strategy on sexually transmitted infections, 2016–2021; 2016 [updated 2016/07//]. Available from: https://www.who.int/reproductivehealth/publications/rtis/ghss-stis/en/.

[4] Wellcome Trust, Boston Consulting Group. Vaccines to tackle drug-resistant infections. An evaluation of R&D opportunities; 2016 [updated 2016]. Available from: https://vaccinesforamr.org/read-the-report/.

[5] Petousis-Harris H., Paynter J., Morgan J., Saxton P., McArdle B., Goodyear-Smith F. (2017). Effectiveness of a group B outer membrane vesicle meningococcal vaccine against gonorrhoea in New Zealand: a retrospective case-control study. Lancet.

[6] Broutet N., Fruth U., Deal C., Gottlieb S.L., Rees H. (2014). participants of the STI Vaccine Technical Consultation. Vaccines against sexually transmitted infections: the way forward. Vaccine.

[7] Gottlieb S.L., Jerse A.E., Delany-Moretlwe S., Deal C., Giersing B.K. (2019). Advancing vaccine development for gonorrhoea and the Global STI Vaccine Roadmap. Sex Health.

[8] World Health Organization. WHO Preferred Product Characteristics (PPCs). Available from: http://www.who.int/immunization/research/ppc-tpp/preferred_product_characteristics/en/.

[9] Kirkcaldy R.D., Weston E., Segurado A.C., Hughes G. (2019). Epidemiology of gonorrhoea: a global perspective. Sex Health.

[10] Hook E.W., Handsfield H.H., Holmes K.K., Sparling P.F., Stamm W.E., Piot P., Wasserheit J.N., Corey L. (2008). Gonococcal infections in the adult. Sex Transm Dis.

[11] Torrone E.A., Morrison C.S., Chen P.L., Kwok C., Francis S.C., Hayes R.J. (2018). Prevalence of sexually transmitted infections and bacterial vaginosis among women in sub-Saharan Africa: An individual participant data meta-analysis of 18 HIV prevention studies. PLoS Med.

[12] Cornelisse V.J., Zhang L., Law M., Chen M.Y., Bradshaw C.S., Bellhouse C. (2018). Concordance of gonorrhoea of the rectum, pharynx and urethra in same-sex male partnerships attending a sexual health service in Melbourne, Australia. BMC Infect Dis.

[13] Brunham R.C., Gottlieb S.L., Paavonen J. (2015). Pelvic inflammatory disease. N Engl J Med.

[14] Haggerty C.L., Gottlieb S.L., Taylor B.D., Low N., Xu F., Ness R.B. (2010). Risk of sequelae after *Chlamydia trachomatis* genital infection in women. J Infect Dis.

[15] Reekie J., Donovan B., Guy R., Hocking J.S., Kaldor J.M., Mak D. (2019). Risk of ectopic pregnancy and tubal infertility following gonorrhea and chlamydia infections. Clin Infect Dis.

[16] Westrom L., Joesoef R., Reynolds G., Hagdu A., Thompson S.E. (1992). Pelvic inflammatory disease and fertility. A cohort study of 1,844 women with laparoscopically verified disease and 657 control women with normal laparoscopic results. Sex TransmDis.

[17] Wiesenfeld H.C., Hillier S.L., Meyn L.A., Amortegui A.J., Sweet R.L. (2012). Subclinical pelvic inflammatory disease and infertility. Obstet Gynecol.

[18] Hillis S.D., Joesoef R., Marchbanks P.A., Wasserheit J.N., Cates W., Westrom L. (1993). Delayed care of pelvic inflammatory disease as a risk factor for impaired fertility. Am J Obstet Gynecol.

[19] Heumann C.L., Quilter L.A.S., Eastment M.C., Heffron R., Hawes S.E. (2017). Adverse birth outcomes and maternal *Neisseria gonorrhoeae* infection: A population-based cohort study in Washington State. Sex Transm Dis.

[20] Laga M., Meheus A., Piot P. (1989). Epidemiology and control of gonococcal ophthalmia neonatorum. Bull World Health Organ.

[21] World Health Organization. WHO guidelines for the treatment of Neisseria gonorrhoeae; 2016 [updated 2016]. Available from: https://www.who.int/reproductivehealth/publications/rtis/gonorrhoea-treatment-guidelines/en/. 27512795

[22] Cohen M.S., Council O.D., Chen J.S. (2019). Sexually transmitted infections and HIV in the era of antiretroviral treatment and prevention: the biologic basis for epidemiologic synergy. J Int AIDS Soc.

[23] Hayes R., Watson-Jones D., Celum C., van de Wijgert J., Wasserheit J. (2010). Treatment of sexually transmitted infections for HIV prevention: end of the road or new beginning?. AIDS.

[24] Sexton J., Garnett G., Rottingen J.A. (2005). Metaanalysis and metaregression in interpreting study variability in the impact of sexually transmitted diseases on susceptibility to HIV infection. Sex Transm Dis.

[25] Johnson L.F., Lewis D.A. (2008). The effect of genital tract infections on HIV-1 shedding in the genital tract: a systematic review and meta-analysis. Sex Transm Dis.

[26] Sonnenberg P., Clifton S., Beddows S., Field N., Soldan K., Tanton C. (2013). Prevalence, risk factors, and uptake of interventions for sexually transmitted infections in Britain: findings from the National Surveys of Sexual Attitudes and Lifestyles (Natsal). Lancet.

[27] Kularatne R.S., Niit R., Rowley J., Kufa-Chakezha T., Peters R.P.H., Taylor M.M. (2018). Adult gonorrhea, chlamydia and syphilis prevalence, incidence, treatment and syndromic case reporting in South Africa: Estimates using the Spectrum-STI model, 1990–2017. PLoS ONE.

[28] Vallely L.M., Toliman P., Ryan C., Rai G., Wapling J., Tomado C. (2016). Prevalence and risk factors of *Chlamydia trachomatis*, *Neisseria gonorrhoeae*, *Trichomonas vaginalis* and other sexually transmissible infections among women attending antenatal clinics in three provinces in Papua New Guinea: a cross-sectional survey. Sex Health.

[29] Werner R.N., Gaskins M., Nast A., Dressler C. (2018). Incidence of sexually transmitted infections in men who have sex with men and who are at substantial risk of HIV infection - A meta-analysis of data from trials and observational studies of HIV pre-exposure prophylaxis. PLoS ONE.

[30] The Kirby Institute. HIV, viral hepatitis and sexually transmissible infections in Australia: Annual surveillance report 2018 | UNSW - The Kirby Institute for infection and immunity in society; 2018.

[31] Centers for Disease Control and Prevention. Sexually Transmitted Disease Surveillance 2018; 2019 [updated 2019/08/27/]. Available from: https://www.cdc.gov/std/stats18/default.htm.

[32] Public Health England. Sexually transmitted infections and screening for chlamydia in England, 2018; 2019 [updated 2019]. Available from: https://assets.publishing.service.gov.uk/government/uploads/system/uploads/attachment_data/file/806118/hpr1919_stis-ncsp_ann18.pdf.

[33] Unemo M., Bradshaw C.S., Hocking J.S., de Vries H.J.C., Francis S.C., Mabey D. (2017). Sexually transmitted infections: challenges ahead. Lancet Infect Dis.

[34] Cates W., Farley T.M., Rowe P.J. (1985). Worldwide patterns of infertility: is Africa different?. Lancet.

[35] Unemo M., Shafer W.M. (2014). Antimicrobial resistance in *Neisseria gonorrhoeae* in the 21st century: past, evolution, and future. Clin Microbiol Rev.

[36] Wi T., Lahra M.M., Ndowa F., Bala M., Dillon J.R., Ramon-Pardo P. (2017). Antimicrobial resistance in *Neisseria gonorrhoeae*: Global surveillance and a call for international collaborative action. PLoS Med.

[37] World Health Organization. Global priority list of antibiotic-resistant bacteria to guide research, discovery, and development of new antibiotics; 2017 [updated 2017/02/27/]. Available from: https://www.who.int/medicines/publications/global-priority-list-antibiotic-resistant-bacteria/en/.

[38] Unemo M. (2015). Current and future antimicrobial treatment of gonorrhoea - the rapidly evolving *Neisseria gonorrhoeae* continues to challenge. BMC Infect Dis.

[39] Fifer H., Natarajan U., Jones L., Alexander S., Hughes G., Golparian D. (2016). Failure of dual antimicrobial therapy in treatment of gonorrhea. N Engl J Med.

[40] Eyre D.W., Sanderson N.D., Lord E., Regisford-Reimmer N., Chau K., Barker L. (2018). Gonorrhoea treatment failure caused by a *Neisseria gonorrhoeae* strain with combined ceftriaxone and high-level azithromycin resistance, England, February 2018. Euro Surveill.

[41] Vallely L.M., Toliman P., Ryan C., Rai G., Wapling J., Gabuzzi J. (2017). Performance of syndromic management for the detection and treatment of genital *Chlamydia trachomatis*, *Neisseria gonorrhoeae* and *Trichomonas vaginalis* among women attending antenatal, well woman and sexual health clinics in Papua New Guinea: a cross-sectional study. BMJ Open.

[42] Wi T.E., Ndowa F.J., Ferreyra C., Kelly-Cirino C., Taylor M.M., Toskin I. (2019). Diagnosing sexually transmitted infections in resource-constrained settings: challenges and ways forward. J Int AIDS Soc.

[43] Mohammed H., Blomquist P., Ogaz D., Duffell S., Furegato M., Checchi M. (2018). 100 years of STIs in the UK: a review of national surveillance data. Sex Transm Infect.

[44] Rice P.A., Shafer W.M., Ram S., Jerse A.E. (2017). *Neisseria gonorrhoeae*: Drug resistance, mouse models, and vaccine development. Annu Rev Microbiol.

[45] Russell M.W., Jerse A.E., Gray-Owen S.D. (2019). Progress toward a gonococcal vaccine: The way forward. Front Immunol.

[46] Fox K.K., Thomas J.C., Weiner D.H., Davis R.H., Sparling P.F., Cohen M.S. (1999). Longitudinal evaluation of serovar-specific immunity to *Neisseria gonorrhoeae*. Am J Epidemiol.

[47] Sánchez-Busó L., Harris S.R. (2019). Using genomics to understand antimicrobial resistance and transmission in *Neisseria gonorrhoeae*. Microb Genom.

[48] Liu Y., Islam E.A., Jarvis G.A., Gray-Owen S.D., Russell M.W. (2012). *Neisseria gonorrhoeae* selectively suppresses the development of Th1 and Th2 cells, and enhances Th17 cell responses, through TGF-β-dependent mechanisms. Mucosal Immunol.

[49] Gulati S., Mu X., Zheng B., Reed G.W., Ram S., Rice P.A. (2015). Antibody to reduction modifiable protein increases the bacterial burden and the duration of gonococcal infection in a mouse model. J Infect Dis.

[50] Paynter J., Goodyear-Smith F., Morgan J., Saxton P., Black S., Petousis-Harris H. (2019). Effectiveness of a group B outer membrane vesicle meningococcal vaccine in preventing hospitalization from gonorrhea in New Zealand: A Retrospective Cohort Study. Vaccines (Basel).

[51] De Wals P., Deceuninck G., Lefebvre B., Tsang R., Law D., De Serres G. (2017). Impact of an immunization campaign to control an increased incidence of serogroup B meningococcal disease in one region of Quebec, Canada. Clin Infect Dis.

[52] Pérez O., del Campo J., Cuello M., Gonzalez E., Nunez N., Cabrera O. (2009). Mucosal approaches in *Neisseria* vaccinology. VacciMonitor.

[53] Beernink P.T., Ispasanie E., Lewis L.A., Ram S., Moe G.R., Granoff D.M. (2019). A meningococcal native outer membrane vesicle vaccine with attenuated endotoxin and overexpressed factor H binding protein elicits gonococcal bactericidal antibodies. J Infect Dis.

[54] Semchenko E.A., Tan A., Borrow R., Seib K.L. (2019). The serogroup B meningococcal vaccine Bexsero elicits antibodies to *Neisseria gonorrhoeae*. Clin Infect Dis.

[55] Connolly KL, Leduc I, Rahman N, Sempowski G, Jerse AE. The group B meningococcal vaccine Bexsero induces antibodies that recognize several candidate gonorrhea vaccine targets and shows protective efficacy against experimental *Neisseria gonorrhoeae* genital tract infection in mice. In Proceedings of the 21st International Pathogenic *Neisseria* Conference; 23–28 September 2018; Pacific Grove, CA, USA; 2018.

[56] Vincent L.R., Jerse A.E. (2019). Biological feasibility and importance of a gonorrhea vaccine for global public health. Vaccine.

[57] Gala R.P., Zaman R.U., D'Souza M.J., Zughaier S.M. (2018). Novel whole-cell inactivated *Neisseria gonorrhoeae* microparticles as vaccine formulation in microneedle-based transdermal immunization. Vaccines (Basel).

[58] Hobbs M.M., Sparling P.F., Cohen M.S., Shafer W.M., Deal C.D., Jerse A.E. (2011). Experimental gonococcal infection in male volunteers: Cumulative experience with *Neisseria gonorrhoeae* strains FA1090 and MS11mkC. Front Microbiol.

[59] Dodet B. (2014). Current barriers, challenges and opportunities for the development of effective STI vaccines: point of view of vaccine producers, biotech companies and funding agencies. Vaccine.

[60] Gottlieb S.L., Giersing B., Boily M.C., Chesson H., Looker K.J., Schiffer J. (2019). Modelling efforts needed to advance herpes simplex virus (HSV) vaccine development: Key findings from the World Health Organization Consultation on HSV Vaccine Impact Modelling. Vaccine.

[61] Craig A.P., Gray R.T., Edwards J.L., Apicella M.A., Jennings M.P., Wilson D.P. (2015). The potential impact of vaccination on the prevalence of gonorrhea. Vaccine.

[62] Heijne J., Xiridou M., Turner K., Van Benthem B., Low N. (2019). The impact of gonorrhoea vaccination in men who have sex with men on prevalence and resistance: Mathematical modelling study. Sex Transm Infect.

[63] Régnier S.A., Huels J. (2014). Potential impact of vaccination against *Neisseria meningitidis* on *Neisseria gonorrhoeae* in the United States: results from a decision-analysis model. Hum Vaccin Immunother.

[64] Yorke J.A., Hethcote H.W., Nold A. (1978). Dynamics and control of the transmission of gonorrhea. Sex Transm Dis.

[65] Garnett G.P. (2014). The theoretical impact and cost-effectiveness of vaccines that protect against sexually transmitted infections and disease. Vaccine.

[66] Owusu-Edusei K., Chesson H.W., Gift T.L., Tao G., Mahajan R., Ocfemia M.C.B. (2013). The estimated direct medical cost of selected sexually transmitted infections in the United States, 2008. Sex Transm Dis.

[67] Chesson H.W., Kirkcaldy R.D., Gift T.L., Owusu-Edusei K., Weinstock H.S. (2018). An illustration of the potential health and economic benefits of combating antibiotic-resistant gonorrhea. Sex Transm Dis.

[68] Korenromp E.L., Wi T., Resch S., Stover J., Broutet N. (2017). Costing of national STI program implementation for the Global STI Control Strategy for the Health Sector, 2016–2021. PLoS ONE.

[69] Centers for Disease Control and Prevention. Antibiotic resistance threats in the United States, 2013; 2013. Available from: https://www.cdc.gov/drugresistance/threat-report-2013/pdf/ar-threats-2013-508.pdf.

[70] Kenyon C., Buyze J., Spiteri G., Cole M.J., Unemo M. (2019). Population-level antimicrobial consumption is associated with decreased antimicrobial susceptibility in *Neisseria gonorrhoeae* in 24 European countries: an ecological analysis. J Infect Dis.

[71] World Health Organization. Value attribution framework for vaccines against antimicrobial resistance; 2019. Available from: https://www.who.int/immunization/research/meetings_workshops/5_Hasso_Prudden_AMR_PDVAC_2019.pdf?ua=1.

[72] Alirol E., Wi T.E., Bala M., Bazzo M.L., Chen X.S., Deal C. (2017). Multidrug-resistant gonorrhea: A research and development roadmap to discover new medicines. PLoS Med.

[73] Chow E.P.F., Walker S., Hocking J.S., Bradshaw C.S., Chen M.Y., Tabrizi S.N. (2017). A multicentre double-blind randomised controlled trial evaluating the efficacy of daily use of antibacterial mouthwash against oropharyngeal gonorrhoea among men who have sex with men: the OMEGA (Oral Mouthwash use to Eradicate GonorrhoeA) study protocol. BMC Infect Dis.

[74] World Health Organization (2017). Human papillomavirus vaccines: WHO position paper, May 2017-Recommendations. Vaccine.

[75] Hoover K.W., Butler M., Workowski K.A., Follansbee S., Gratzer B., Hare C.B. (2012). Low rates of hepatitis screening and vaccination of HIV-infected MSM in HIV clinics. Sex Transm Dis.

[76] Villena R., Safadi M.A.P., Valenzuela M.T., Torres J.P., Finn A., O'Ryan M. (2018). Global epidemiology of serogroup B meningococcal disease and opportunities for prevention with novel recombinant protein vaccines. Hum Vaccin Immunother.

[77] Gottlieb S.L., Deal C.D., Giersing B., Rees H., Bolan G., Johnston C. (2016). The global roadmap for advancing development of vaccines against sexually transmitted infections: Update and next steps. Vaccine.

[78] Global Vaccine Action Plan (2013). Decade of vaccine collaboration. Vaccine.

[79] World Health Organization. Global Vaccine Action Plan 2011–2020; 2012 [updated 2012]. Available from: http://www.who.int/immunization/global_vaccine_action_plan/GVAP_doc_2011_2020/en/.

[80] Every Woman Every Child. Global Strategy for Women's, Children's and Adolescents Health 2016–2030; 2015 [updated 2015]. Available from: https://www.who.int/life-course/publications/global-strategy-2016-2030/en/.

[81] World Health Organization. Global action plan on antimicrobial resistance; 2015 [updated 2015]. Available from: https://apps.who.int/iris/bitstream/handle/10665/193736/9789241509763_eng.pdf;jsessionid=96D175C723729F840B5C008ADBD609B7?sequence=1. 10.7196/samj.9644 26242647

[82] World Health Organization. Global action plan to control the spread and impact of antimicrobial resistance in *Neisseria gonorrhoeae*. Available from: https://www.who.int/reproductivehealth/publications/rtis/9789241503501/en/.

[83] Wetzler L.M., Feavers I.M., Gray-Owen S.D., Jerse A.E., Rice P.A., Deal C.D. (2016). Summary and recommendations from the National Institute of Allergy and Infectious Diseases (NIAID) workshop “Gonorrhea Vaccines: the Way Forward”. Clin Vacc Immunol.

[84] Toskin I., Murtagh M., Peeling R.W., Blondeel K., Cordero J., Kiarie J. (2017). Advancing prevention of sexually transmitted infections through point-of-care testing: target product profiles and landscape analysis. Sex Transm Infect.

[85] World Health Organization. Standard protocol to assess prevalence of gonorrhoea and chlamydia among pregnant women in antenatal care clinics; 2018 [updated 2018]. Available from: http://www.who.int/reproductivehealth/publications/rtis/gonorrhoea-chlamydia-among-pregnant-women/en/

[86] Geisler W.M., Wang C., Morrison S.G., Black C.M., Bandea C.I., Hook E.W. (2008). The natural history of untreated *Chlamydia trachomatis* infection in the interval between screening and returning for treatment. Sex Transm Dis.

[87] Geisler W.M., Lensing S.Y., Press C.G., Hook E.W. (2013). Spontaneous resolution of genital *Chlamydia trachomatis* infection in women and protection from reinfection. J Infect Dis.

[88] Crew P.E., Abara W.E., McCulley L., Waldron P.E., Kirkcaldy R.D., Weston E.J. (2018). Disseminated gonococcal infections in patients receiving eculizumab: a case series. Clin Infect Dis.

